# Removal of 2‐Rod Subdermal Implant Identified on X‐Ray: A Case Report

**DOI:** 10.1002/ccr3.70737

**Published:** 2025-08-07

**Authors:** Laura C. D. Pomatto‐Watson, Julia Hutchison, Justin T. Hutchison

**Affiliations:** ^1^ School of Medicine University of California, Davis Sacramento California USA; ^2^ Kaiser Permanente Modesto Medical Center Modesto California USA

**Keywords:** Jadelle, long‐acting reversible contraception, Nexplanon, subdermal implant

## Abstract

Case report of a 37‐year‐old Colombian patient seeking removal of a subdermal implant, initially believed to be the single‐rod Nexplanon implant. However, following extraction, it was identified as the 2‐rod international subdermal implant alternative, Jadelle. This case highlights the need for US health care providers to be cognizant of non‐US subdermal alternatives given the rising presence of foreign‐born patients they may encounter in their practice.

## 1 Introduction

Subdermal hormonal contraceptive implants afford long‐term reversible pregnancy protection that is highly effective and safe while maintaining reproductive autonomy [1]. Often termed the “forgettable” contraception as its efficacy is independent of user adherence [2], which is unlike the once‐daily pill, quarterly injections, or barrier protection methods [3]. The efficacy of subdermal implants is on par with other forms of long‐acting contraception (LARCs), including intrauterine devices (IUDs) and permanent sterilization, with an annual pregnancy rate < 1% [4], and the added benefit of not requiring a pelvic exam for placement.

Subdermal contraceptive implants exert their effect via progestin, which inhibits ovulation and promotes barrier protection via thickening of the cervical mucus [5]. Norplant was the first contraceptive implant—comprised of 6 rods inserted in the upper arm, with each rod containing 36 mg of levonorgestrel and shown to be highly effective. It was removed from the US market in 2002 due to complications with extraction [5]. Norplant, however, remained readily available worldwide until its discontinuation in 2008 [6] due to increasing favor for second‐generation subdermal implants. Nexplanon is the only FDA‐approved radiopaque single‐rod etonogestrel 3‐year implant currently available in the US [3] and is the predominant device US physicians are trained to recognize and use. Studies also demonstrate continued effectiveness through 5 years of use [7].

Yet, Nexplanon is inaccessible to women outside the US. Worldwide, a major barrier to accessing long‐term contraception, including subdermal implants, is the high upfront cost. As a result, < 10% of women in Latin American countries rely on long‐acting contraception [8] and globally, < 1% of reproductive‐age women rely on subdermal implants [6]. Those who can access subdermal implants rely on 2 equally effective alternatives to Nexplanon: Implanon and Jadelle [9, 10]. Implanon is Nexplanon's radio‐translucent predecessor, consisting of a single rod with 68 mg etonorgestrel with a 3‐year duration of efficacy [11], whereas Jadelle is a 2‐rod implant, with each rod containing 75 mg levonorgestrel, with a 5‐year duration of efficacy [6]. Until recently, Jadelle and Implanon cost, on average, $20–22—an insurmountable price for many low‐ to middle‐income countries and donor agencies [6]. Because of the relatively low usage rate of non‐US‐available subdermal implants, coupled with the unique insertion and removal aspects of Jadelle (rods form a 30° angle upon insertion, requiring a modified U‐technique upon removal) [5], many US providers are unfamiliar with the 2‐rod alternative.

Limited US‐physician training regarding Nexplanon 2‐rod alternatives has potentially high reproductive consequences for foreign‐born women seeking device removal. Over 14% of the US population is foreign‐born, with the majority being reproductive‐age adults [12]. Incorrect removal (i.e., only 1 of the 2 rods) may result in incorrect inferility etiology given prolonged pregnancy protection beyond approved shelf life [13]. Thus, physicians caring for reproductive‐age patients need to be cognizant of non‐US contraceptive alternatives, as correct identification of a reversible cause of infertility may assuage costly, time‐consuming infertility workup.

In this report, we present a case of incorrect identification of the type of subdermal contraceptive implant (1‐rod vs. 2‐rod device) that highlights the necessity of US physicians to consider country‐dependent access to the types of subdermal contraceptive implants.

## 2 Case History & Examination

A 37‐year‐old G1P1 Spanish‐speaking patient presented to an OBGYN outpatient clinic for removal of a subdermal contraceptive implant. The patient was unable to recall the type of implant but believed it to be Nexplanon and shared it had been inserted in Colombia approximately 4 years prior.

During the initial removal attempt, the implant was palpated and visualized via skin tenting in the left arm 4 cm above the medial epicondyle. The patient's skin was prepped with alcohol and followed with a subdermal injection of 1% lidocaine at the site of skin tenting. A 3 mm incision was made, and despite palpation of the implant and visualization on ultrasound, the implant could not be removed as it appeared to be bent. Prior cases have reported similar occurrences in atraumatic settings [14]. Due to the concern the implant was beyond the FDA‐approved shelf life (3 years), the initial provider was concerned about the increased risk of fibrotic capsule formation around the implant [15] or migration of the implant into deeper tissue [16], which could further impede extraction in the outpatient setting. The patient was advised to return for ultrasound‐guided removal.

## 3 Differential Diagnosis, Investigation, and Treatment

**FIGURE 1:**
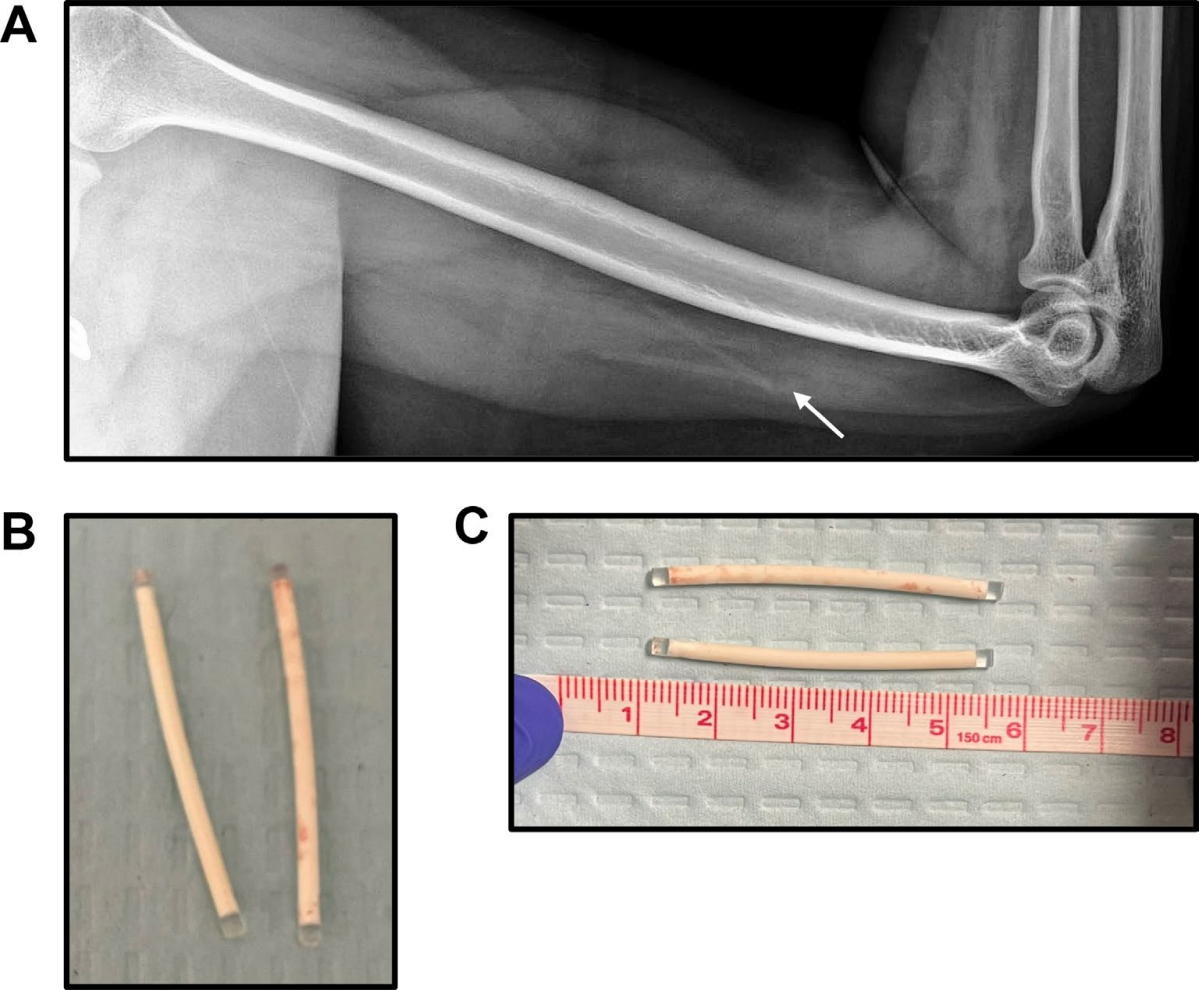
(A) X‐ray of subdermal implant prior to removal. (B and C) Visual inspection of the 2‐rod implant following removal.

Ultrasound imaging of the left upper extremity showed possible 2 echogenic linear implants. It was unclear if the implant had been broken into 2 pieces (~2 cm × 2 cm) as prior cases reported with Nexplanon [14] or was possibly a 2‐rod implant. The patient was unable to recall if multiple implants had been inserted. A follow‐up x‐ray of the left humerus indicated two faint cylindrical densities (Figure 1A), suggestive of a 2‐rod implant, possibly Jadelle, due to the initial country of insertion. The location of the implants was confirmed via ultrasound, and a 1% lidocaine solution was injected deep to the implants. A 3 mm skin incision was made. Using ultrasound to help guide retrieval, 2 intact implants were successfully removed (Figure 1B,C).

## 4 Conclusion and Results

Successful identification of the type of subdermal implant enabled out‐patient extraction and avoided unnecessary surgical intervention and decreased burden on the patient (i.e., additional medical expense, surgical time, and anesthesia). The present report demonstrates the importance of correctly identifying the type of subdermal contraceptive implant. Incorrect identification can result not only in unnecessary surgical procedures but may inadvertently result in an unknown cause of infertility. This case report highlights the necessity of US care providers to be adequately trained in the types of globally available subdermal contraception. Health care providers need to be familiar with the types of contraception predominantly available in their patient's country of origin/location of initial insertion. Incorrect removal of subdermal contraceptive implants can result in unanticipated infertility consequences.

## 5 Discussion

Here, we present a case of incorrect identification of Nexplanon‐alternative subdermal implant (1‐rod vs. 2‐rod device). To our knowledge, misidentification of the type of subdermal contraceptive implant has previously not been reported. This finding is important as it highlights how incomplete removal may lead to iatrogenic infertility. Correct identification of the type of subdermal contraceptive implant is imperative for appropriate extraction in the out‐patient setting. The 2‐rod implant requires utilization of the “U technique,” which relies upon modified vas deferens holding forceps to grasp the implants from the side rather than the tip [17], as done with the single‐rod implant [18]. In turn, this decreases the risk of implant breakage [19] or the need for more extensive and costly surgical intervention.

It is important to highlight in the present case the initial concern that the implant was bent or broken, which would render it ineffective [20]. Clinical reports on damaged single‐rod subdermal implants are limited. The majority of cases are attributed to patient‐derived causes—including palpation, strenuous upper body exercises, or trauma to the implant site [5, 21], but more recent reports also indicate non‐traumatic damage [14, 22]. Additional complications arise if the implant becomes impalpable, often requiring surgical extraction [23, 24] or concern for implant migration to precarious locations [16, 25, 26]. Similar complications have been reported for the 2‐rod device but are mainly associated with inappropriate extraction techniques [19].

Subdermal hormonal contraceptive implants are highly effective reversible contraception with very low failure rates (< 1%) rivaling that of permanent sterilization [3]. Conversely, resumption of fertility is high following implant removal, with ovulation reportedly resuming within 6 weeks and estimated fertility post‐implant removal (76.55%–85.6%) matching those of women not using contraception (82% rate of pregnancy within 1 year of unprotected sex) [27]. Together, these findings indicate subdermal implants perform exceptionally well in their intended purpose: to prevent pregnancy. But unlike more invasive and irreversible approaches, these effects are easily reversible, enabling patients to maintain their reproductive autonomy.

However, to help patients maintain their reproductive freedom, US physicians need to be cognizant of patients' country of origin for predominant types of subdermal contraceptive implants. Patients who wish to become pregnant may face unintended infertility complications if rods of subdermal implants are inadvertently left in place. For example, Jadelle was found to be efficacious for nearly 2–7 years beyond its approved lifespan [13], with increased rates of pregnancy dependent on increasing BMI. Patients who are unfamiliar with the type of implant they received, especially outside the US, may inadvertently face a reversible, albeit difficult to identify, cause of infertility.

As 1 in 6 US citizens will be foreign‐born by 2060 [12], the necessity of healthcare providers trained in all globally available types of subdermal implants is imperative. To remediate this gap in knowledge, clinicians should familiarize themselves with multiple extraction techniques, providing them with additional tools for successful out‐patient removal [28, 29, 30]. Alternatively, some institutions have introduced family planning specialty referral centers [31, 32], further alleviating the need for more extensive surgical intervention for implant extraction.

## Author Contributions

**Laura C. D. Pomatto‐Watson:** conceptualization, formal analysis, writing – original draft, writing – review and editing. **Julia Hutchison:** writing – review and editing. **Justin T. Hutchison:** conceptualization, data curation, formal analysis, project administration, supervision, writing – original draft, writing – review and editing.

## Consent

Written informed consent was obtained from the patient prior to publication of this case report.

## Conflicts of Interest

The authors declare no conflicts of interest.

## Data Availability

Data available on request due to privacy/ethical restrictions.
